# The Temporal Associations of Neck Pain and Headache – Implications for the Diagnostic Approach to the Myofascial Involvement in Migraine

**DOI:** 10.1177/11795735251404279

**Published:** 2025-12-11

**Authors:** Corinna Börner-Schröder, Thomas Lachhammer, Paula Behrendt, Theresa Pfeifer, Paulina Kolorz, Sarah Lense, Julie Pompignoli, Miriam Reichert, Severin Schramm, Florian Heinen, Nico Sollmann, Michaela V. Bonfert

**Affiliations:** 1 MUC – Munich University Center for Children with Medical and Developmental Complexity – MUC Hauner, LMU University Hospital, LMU Munich, Munich, Germany; 2 Division of Pediatric Neurology and Developmental Medicine, Department of Pediatrics, Dr. Von Hauner Children’s Hospital, LMU University Hospital, Munich, Germany; 3 Department of Diagnostic and Interventional Neuroradiology, School of Medicine and Health, Klinikum rechts der Isar, Technical University of Munich, Munich, Germany; 4 TUM-Neuroimaging Center, Klinikum rechts der Isar, Technical University of Munich, Munich, Germany; 5 Department of Diagnostic and Interventional Radiology, University Hospital Ulm, Ulm, Germany

**Keywords:** primary headache disorder, pediatric headache, ultrasound, upper trapezius muscle, pressure pain threshold, myofascial trigger point

## Abstract

**Background:**

Migraine remains a relevant source of disability. Peripheral pathophysiological mechanisms including the involvement of neck musculature are not yet well understood. A temporal association of headache and neck pain, and imaging tools for its assessment are not established.

**Objectives:**

Our aim was to explore the association between headache episodes and involvement of neck muscles in patients with episodic migraine and healthy controls.

**Design:**

Controlled clinical study.

**Methods:**

Data of 13 migraine patients (26.92 ± 2.47 years, 12 females) and 13 matched healthy controls (26.62 ± 3.43 years) on headache, migraine, and neck pain were collected during an initial 12-week observational period. A cross-sectional examination followed that comprised clinical assessment of the upper trapezius muscle (UTM) including identification of myofascial trigger points (mTrP), algometry (pressure pain thresholds [PPT]), and B-mode (brightness mode) ultrasound measurements of muscle and fascia thickness and gray scale analysis.

**Results:**

Migraine patients reported significantly higher neck pain frequency and duration and significantly lower PPT above the UTM (*P* < 0.05) than controls. Mean PPT values of mTrP in patients did not significantly differ from PPT values of reference points on the same side (left: *P* = 0.419, right: *P* = 0.100). The odds ratio of co-occurring headache or migraine on days with neck pain was 5.64 (95% confidence interval [CI] [4.14;7.69]) and 7.21 (95% CI [4.95;10.49]) times higher than on neck pain-free days. Ultrasound analysis demonstrated significant differences in muscle/fascial thickness in 12 out of 24 measurements. There were no significant differences in gray scale analysis between groups. When comparing same-side ultrasound measurements of pooled reference points and mTrP, all measurements of muscle thickness (*P* = 0.002, 0.006, 0.002, 0.012), one measurement of fascial thickness (*P* = 0.006), and three measurements of gray scale (*P* = 0.009, 0.014; *P* < 0.001) yielded significant results.

**Conclusions:**

Our data may emphasize the relevance of UTM myofascial involvement in migraine patients. This may involve the UTM as a whole, rather than single focalities. The muscular component of migraine and other headache disorders remains an overlooked part of diagnosis and treatment. Consequently, imaging methods, especially low-cost point of care tools such as ultrasound, may provide objectifiable additional data to known clinical findings.

**Registration:**

Clinical trial registration: DRKS (German Clinical Trials Register): “Neuromodulation by stimulation of cervical afferents in migraine patients - the neurophysiological basis of repetitive peripheral magnetic stimulation (rPMS) in patients with episodic migraine” ID: DRKS00024470, https://drks.de/search/en/trial/DRKS00024470/entails.

## Introduction

Primary headaches are an entity affecting a relevant part of the general population.^1^ In particular, migraine represents a meaningful source of disability and overall loss of quality of life.^1^ Young adults are among the most affected individuals with a peak of incidence reached for children and adolescents aged 10-14 years.^2^ While migraine is generally known for headache as the leading symptom, a mere focus on central mechanisms underappreciates the complexity of the pattern of symptoms and the related pathophysiology.

The coexistence of central and peripheral mechanisms involved in pain generation, processing, perception, and perpetuation is well accepted. In this context, the trigemino-vascular system is one of the key players.^3^ Another key role plays the trigemino-cervical complex (TCC), which links myofascial symptoms and muscular findings of the pericranial and neck region to migraine pathophysiology.^4^ The TCC hypothesizes that nociceptive and proprioceptive input via Aδ and C fibers from pericranial and neck muscles is referred through convergence with sensory fibers of the first trigeminal branch.^5^ This happens at the level of the spinal trigeminal nucleus and signals are forwarded as well as integrated into higher pain processing pathways.^4^ This relay of nociceptive information is aggravated by an increased responsiveness of the myofascial nociceptors and the neurons in the trigeminal ganglion caused by a mechanism referred to as peripheral sensitization.^6-13^ In this regard, myofascial peripheral sensitization continuously exaggerates and perpetuates, which can lead to pain perceived in the neck area in addition to headache.

This bottom-up connection has led to an increased interest in the possible role of alterations of the neck musculature regarding the individual patterns of migraine of patients, partially because of the frequent occurrence of cranio-cervical symptoms in patients affected by primary headaches.^14-19^ It has been reported that during migraine attacks, neck pain was even more common than nausea.^20^ Additionally, pathological findings regarding the cervical spine and several cranio-cervical muscles including the upper trapezius muscle (UTM) were observed in migraine patients compared to healthy controls.^10-12,21^ Moreover, neck muscles of migraine patients are more sensitive to pressure and often contain myofascial trigger points (mTrP) as common focal findings.^22-25^ Specifically, an mTrP was first defined by Travell and Simons as a “hyperirritable spot in a skeletal muscle that is associated with a hypersensitive palpable nodule in a taut band.^26^ The spot is painful on compression and can give rise to characteristic referred pain, referred tenderness, motor dysfunction and autonomic phenomena”.^26,27^Hence, Fernandez et al found that mTrP are more frequent in migraine patients than in healthy controls.^23^ Specifically, migraine patients were shown to have a significantly higher number of active, but not latent mTrP in muscles of the cranio-cervical region (UTM, sternocleidomastoid, temporalis, and suboccipital muscles).^23^

Although muscular symptoms and myofascial findings are common in migraine patients and there is a possible explanation for a causal link represented by the TCC, there is no conclusive evidence as to whether symptoms and findings in the neck region are a cause or a consequence of migraine headaches.^28^ So far, the reference standard for assessing muscular symptoms in migraine patients remains a clinical examination including manual palpation.^29^ However, quite recently, pilot imaging studies succeeded to visualize focal alterations at the sites of manually diagnosed active mTrP,^30^ and revealed overall differences in tissue composition of the UTM in migraine patients using advanced quantitative magnetic resonance imaging (MRI).^30-32^ Specifically, muscle T2 values, potential surrogate measures of water content in tissue, were significantly elevated in the UTM of migraine patients.^30-32^ Other non-invasive imaging techniques under investigation are for instance infrared thermography and muscular ultrasound.^22,33^

In the available studies involving B-mode (brightness mode) imaging, mTrP have been described as hypoechoic spots or regions with a more heterogeneous structure than muscular tissue surroundings.^33,34^ It is hypothesized that these visually detected alterations are the equivalent of local tissue edema, possibly caused by local inflammatory processes.^34^ Gray scale analysis is a method widely used in the field of neuromuscular disorders.^35^ It can be used to assess texture by assigning every pixel in a sonogram an individual numeric value as an approximation of echogenicity, and then differences within or between structures can be analyzed.^36^ For the detection of mTrP, gray scale analysis has not yet been widely used. Takla et al showed a detection accuracy of 33% for active and 35% for latent mTrP in patients with lower back pain (M. quadratus lumborum, M. longissimus thoracis, M. piriformis, and M. gluteus medius muscles).^37^ Consistent with the visual assessment, identified mTrP were found to be hypoechoic compared to the uniform echogenicity of the surrounding muscular tissue.^37^ Nonetheless, B-mode ultrasound as an established, low-cost, non-invasive point-of-care diagnostic tool is a method that warrants further research in the field of migraine. This could include additional outcome measures like muscle and fascia thickness, but also measurements not limited to areas of distinct mTrP but rather of the whole muscle to detect correlates of changes of the overall muscular composition.

Against this background, the aim of this work was to investigate differences in muscular symptoms and myofascial findings in a cohort of migraine patients as compared to healthy individuals, including possible associations of headaches/migraine attacks and neck pain in the migraine group over time. In addition, findings of myofascial assessment of the UTM including manual examination, algometry, and muscular ultrasound were compared between the 2 groups to explore if clinically relevant differences exist.

## Methods

### Ethics

This prospective study was approved by the ethic committees of the Ludwig-Maximilian-University and the Technical University of Munich (vote numbers 20-0575 [LMU Munich], 2020-370_4-S-KH [TU Munich]). The trial was registered with the German Clinical Trials Register (DRKS, ID: DRKS00024470).

It was conducted in accordance with the World Medical Association’s Declaration of Helsinki. All participants provided written informed consent before being enrolled in the study.

### Study Design

Individuals affected by episodic migraine and healthy participants were recruited via postings on the premises of the local university hospitals, universities, as well as different facilities of student services.

Sex, age and body mass index (BMI) matching was done for groups with a tolerance of less than 1 standard deviation (SD). After initial screening for eligibility, participants of both groups were asked to keep headache and neck pain calendars over an observational period of 12 weeks. This longitudinal approach built the basis for the collection of data on a headache-neck pain association. After the observational period, a single appointment for the cross-sectional data collection followed. This assessment included physical examination of the UTM of both sides including the identification of latent and active mTrP, algometry, and B-mode ultrasound. None of the patients reported having a migraine on the day of assessment. The succession of examinations was structured in the following way to minimize changes induced by manual manipulation: starting with manual palpation for mTrP identification, followed by an adjustment period of 15 minutes, subsequent ultrasound and lastly PPT measurements. A complete outline of all outcome measures is summarized in Supplemental Table 1.

### Participants

Inclusion criterion for both groups was age of 18-35 years. In addition, the inclusion criteria for migraine patients were: (I) physician-diagnosed migraine for at least 6 months according to the International Classification of Headache Disorders third edition (ICHD-3) criteria, with at least 5 headache days per month and without fulfilling criteria for chronic migraine, and (II) at least one active or latent mTrP in the UTM of either side according to manual palpation during the screening assessment. Exclusion criteria for migraine patients were: (I) a diagnosis of familial hemiplegic migraine, (II) intake of preventive medication (except for magnesium), and (III) any prior surgery to the shoulder/neck region. Candidates for both groups were not eligible to participate in the study if they (I) were affected by any other neurologic or psychiatric disorders (except for migraine or migraine + tension-type headache (TTH) in the patient group), or (II) other serious preexisting conditions (such as malignant or chronic inflammatory diseases). Study duration for the migraine group was from November 2021 to April 2023, for the control group it ranged from April 2021 to January 2023.

### Documentation of Headache and Neck Pain

All participants were asked to document their headaches via a headache calendar issued by the German Migraine and Headache Society for a period of 12 weeks (84 days).^38^ When suffering from acute headache, participants were instructed to record pain characteristics such as duration, perceived intensity on a 10-point numerical rating scale (NRS), pain localization, accompanying symptoms, and medication, if used. This allowed us to record information such as average number of headache attacks, pain duration, and pain intensity.

Based on this information, we characterized headache episodes into migraine and non-migraine headache days using the following criteria outlined by Tassorelli et al in accordance with ICHD-3 criteria: headache attacks lasting 4-72 hours (untreated or unsuccessfully treated), and headache had at least 2 of the following characteristics: (I) unilateral location, (II) pulsating quality, (III) moderate to severe pain intensity and during headache at least one of the following symptoms was present: (A) nausea and/or vomiting, (B) photophobia or phonophobia.^39^ Headaches were also rated as migraine when they lasted shorter than 4 hours but had been successfully treated with migraine-specific acute medication.^39^ Two examiners (CBS, TL) independently rated the headache episodes as migraine headache or non-migraine headache while being blinded to the results of each other. Whenever there was dissent on whether a headache was to be categorized as migraine or not, a clinician with 12 years of experience (MVB) in clinical care of and research for headache disorders made an expert decision.

Participants also recorded their neck pain including neck pain intensity (on the 10-point NRS), duration, localization, and accompanying symptoms in similar calendars. Thereby, headache and neck pain could be compared day-by-day and temporal relationships could be investigated over the 12-week period.

### Headache Characteristics

At the cross-sectional assessment (after the 12-week assessment period), every participant of the migraine group was personally interviewed to assess for their individual migraine and headache symptomatology. This included the reconfirmation of their specific headache diagnosis, age of onset, typical symptoms, and the acute medication they typically take for treatment of headaches.

### Physical Examination of the UTM

At the cross-sectional assessment every participant’s neck musculature was thoroughly clinically and manually investigated by a board-certified physiotherapist with 6 years of clinical experience. Over the 2 days prior to the assessment day, participants were instructed not to manipulate or exercise their shoulder/neck musculature in any way. The three diagnostic criteria of mTrP were set as follows: (I) a hypersensitive spot in (II) a taut band with III) a referred sensation upon palpation.^27^ When the referred sensation was experienced like the participant’s migraine headache, this mTrP was classified as an active mTrP, and all other mTrP were classified as latent mTrP.^27^ If several mTrP on one body side were detected, only the subjectively most painful one of each side was selected and its position was recorded. If active and latent mTrP on the same side were detected the active one was recorded.

### Pressure-pain Thresholds of the UTM

**Figure 1.**
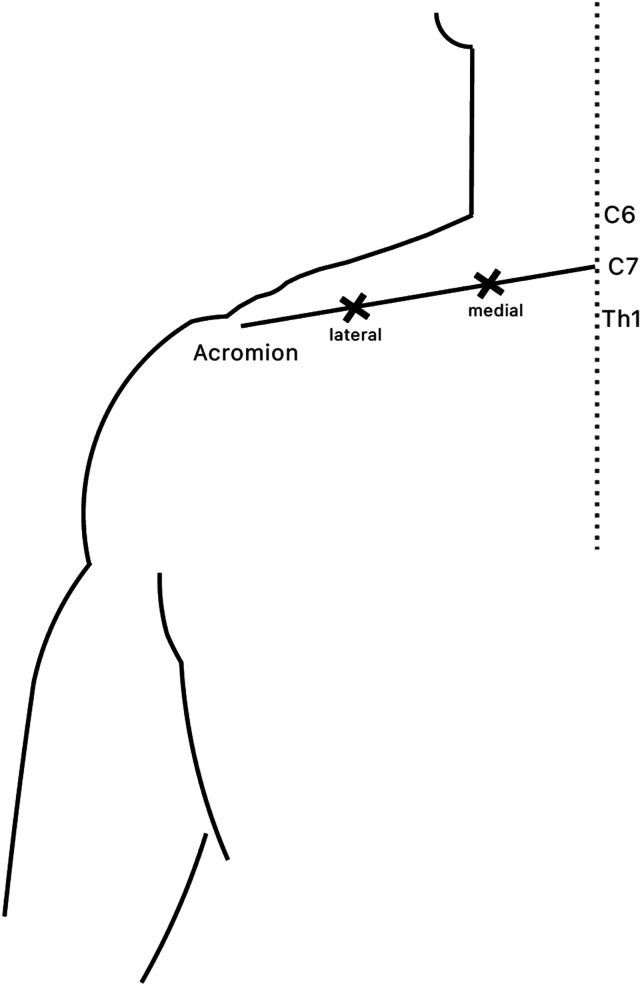
Visualization of Two Reference Points at 1/3 and 2/3 of the Axis Between C7 and Acromion on the Left Side, Seen From Posterior (Marked With x)

On each body side above the bilateral UTM, 2 reference points were marked at 1 (“medial”) and 2 thirds (“lateral”) of the connecting axis between vertebra C7 and the acromion (Figure 1). Using an algometer (Pain Diagnostic and Treatment (pdt); Algomed, Medoc Ltd., Ramat Yishai, Israel), PPT above these reference points as well as above the marked mTrP, if present, were manually measured. Specifically, PPT were determined by consistently increasing pressure of about 0.3 kg/cm^2^/s until the participants verbally signaled that the PPT was reached. Participants were blinded towards their individual results, as not to encourage voluntarily enduring higher levels of pressure. Measurement of each reference point and the most painful mTrP, if detected, was repeated thrice, and a mean value for each point was calculated. This led to a maximum number of three points of measurement for each side, if an mTrP was detected beforehand.

### B-Mode Ultrasound

To assess for possible tissue alterations of the UTM, B-mode ultrasound was performed above the 2 reference points and the most painful manually detected mTrP per body side, if present. Corresponding to the PPT measurements, a maximum number of three points were recorded for each side. We used a 15-6 MHz HFL50xp probe (Fuji Sonosite X-Porte; FUJIFILM Sonosite Inc., Bothell, WA, USA) and the preset program “musculoskeletal examination”. The examiner positioned the ultrasound probe’s indicator directly on the center of the reference point or mTrP, respectively. Probes were held in a 90° angle to the body surface underneath and without application of pressure to correctly record the muscle’s vertical dimension. Similar to PPT measurements, ultrasound recordings were repeated thrice for each point in both longitudinal and transversal orientation.

**Figure 2.**
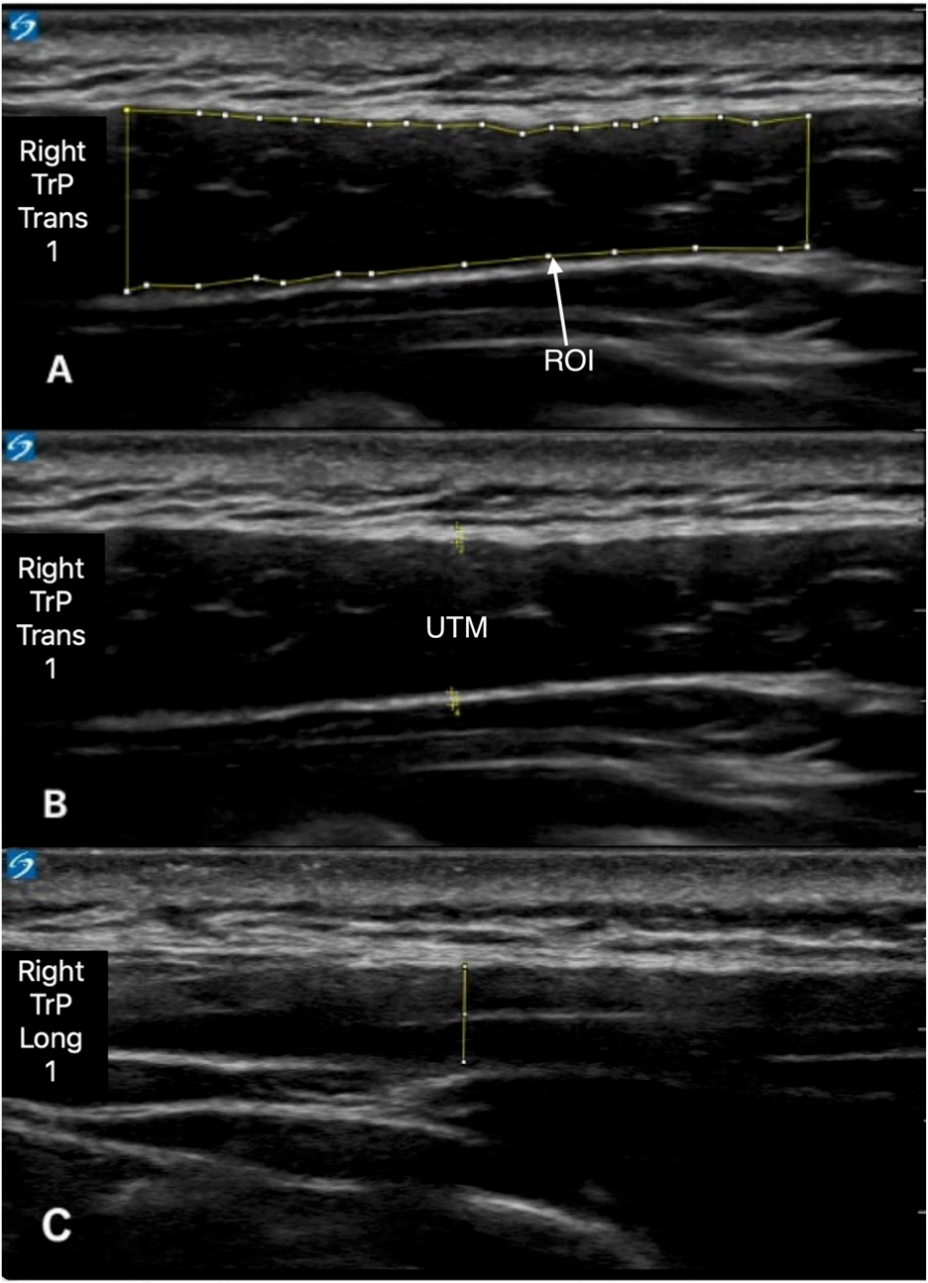
Ultrasound Image of the UTM [Upper Trapezius Muscle] in Transversal (A + B) and Longitudinal (C) orientation A: Selection of the ROI [Region of Interest] for Gray Scale Analysis; ROI Were Set by Selecting the UTM Muscular Tissue in the Approximated Central 75% of the Picture’s width. B: Measurement of Upper and Lower Fascial thickness. C: Measurement of Muscle Belly Thickness (Image Editing by FIJI Version 1.54f). Abbreviations: TrP (Myofascial Trigger Point), Trans (Transversal), Long (Longitudinal)

The visual analysis of the acquired ultrasound images was performed using FIJI (https://imagej.net/software/fiji/, Version 1.54f^40^). Thereby, we measured muscle thickness, as well as upper and lower fascia thickness of the UTM. The examiner determined the horizontal center of each sonogram for these measurements unless that was not possible due to, for instance, poor image quality. Thus, all measurements to determine the thickness of the muscle and the fascia were recorded on the exact midpoint of each sonographic image, as this represents the closest approximation of the individual point targeted by the ultrasound probe. For fascia measurements, the objective was to measure the thickness of the fascial structure in direct contact with the muscular tissue of the UTM’s muscle belly. Because of the filamentous nature of the fibrous tissue, this was defined as the hyperechogenic structure closest to the muscle.^41^ Measurement of muscle and fascia thickness is exemplified for both transversal and longitudinal directions in Figure 2.

For gray scale analysis, a region of interest (ROI) was set, encompassing the muscular tissue occupying approximately 75% of the ultrasound image’s width around the most central point (Figure 2). The borders were put around the inner fascial margins, while not including any part of the hyperechoic fascia itself.^42^ Gray scale values were extracted in a numeric range from 0 to 255. For this purpose, images were converted to 8-bit and mean, minimum, and maximum gray scale values, as well as SD were registered.^36,43^ Due to missing footage, complete ultrasound datasets were only extractable for 12 patients for muscle thickness and 11 patients for gray scale analysis, respectively.

### Statistical Analysis

All data were recorded on paper forms and digitized in Excel sheets (Microsoft Excel 2016, Microsoft Corp. (2016), Redmond, WA, USA). Statistical analysis was performed using SPSS (version 29.0; IBM SPSS Statistics for Windows, IBM Corp., Armonk, NY, USA). The level of statistical significance was set at *P* < 0.05. Descriptive values in tables are given for all included subjects, inclusion in statistical tests may differ slightly.

We performed Shapiro-Wilk tests for assessing data distribution in all numerical categories. An overview of outcome measures and statistical tests is given in Supplemental Table 1. Comparisons between the migraine and control group were performed for age and BMI using Mann-Whitney-U tests, headache frequency, medication intake frequency, and neck pain frequency using Wilcoxon tests and for headache intensity, headache duration, neck pain duration, and neck pain intensity using paired samples t-tests.

For the analysis of daily events, every single day of the observational period of 12 weeks for every migraine patient was analyzed. This included headache frequency, headache intensity, whether acute medication was taken on that day, neck pain frequency, neck pain intensity, and the rating as a migraine/non-migraine headache. For daily superposition of migraine, headache and neck pain days, the calendars’ data of three subjects were not included in this analysis due to insufficient data (did not return complete neck pain calendars). To test the association of headache and neck pain events over time, Chi-squared tests and odds ratios (OR) were calculated. To check for differences in headache characteristics on neck pain days vs non-neck pain days, we performed Wilcoxon tests comparing headache intensity and duration on days with and without neck pain.

**Figure 3.**
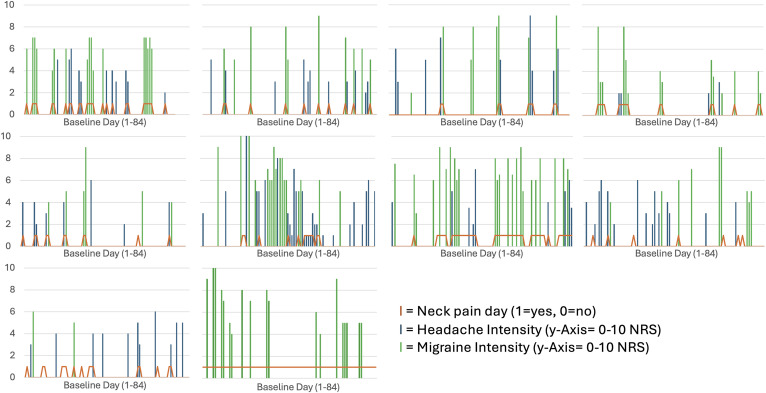
Illustration of the Individual Superposition of Migraine Intensity, Non-migraine Headache Intensity, and Neck Pain Occurrence in 10 Migraine Patients. Blue/Green Pillars Mark the Headache Intensity on a Particular Headache Day. Orange Lines Mark the Occurrence of Neck Pain in a Binary Manner (With 0 Indicating No Neck Pain and Increases to 1 Indicating the Presence of Neck Pain). Graphs are Sorted by Percentage of Neck Pain Days With Co-occurring Headache (From High to Low) From the Upper Left to the Lower Right Graph. Abbreviations: NRS (Numerical Rating Scale)

For comparison of PPT measurements between the migraine and control group, paired samples t-tests or Wilcoxon tests were used. Moreover, in migraine patients, PPT measured above mTrP were compared with the mean PPT values of the reference points of the same side using paired samples t-tests.

Regarding ultrasound data, comparisons of muscle and fascia thickness for all reference points as well as gray scale data (mean, minimum, maximum, and SD) between the migraine and control group were performed using Wilcoxon tests and paired samples t-tests (depending on normality of data distributions). Muscle and fascia thickness were analyzed as a ratio of thickness/BMI in order to account for a subject’s individual body composition. In analogy to PPT measurements, the pooled ultrasound data of reference points (quotients of muscle thickness, upper and lower fascia thickness, gray scale values) were compared to those of mTrP of the ipsilateral UTM using either Wilcoxon tests or paired samples t-tests. Correction for multiple testing was done using the Benjamini-Hochberg procedure.

## Results

### Subject Characteristics

The study cohort included 13 patients with episodic migraine and 13 sex-matched, healthy controls. Migraine and control groups did not significantly differ in age (migraine group: 26.92 ± 2.47 years, control group: 26.62 ± 3.43 years, *P* = 0.479), sex (12 females, 1 male), or BMI (migraine group: 25.07 ± 3.77 kg/m^2^, control group: 22.81 ± 2.96 kg/m^2^, *P* = 0.057, Table 1). Overall, 12 participants in the migraine group and all 13 participants in the control group were right-handed. 66.6% of female migraine patients and 58.3% of female healthy controls used hormonal contraception. All participants met general university entrance qualification with an “Abitur” (German A-level-equivalent) or held a completed university degree. 62% of participants in both groups were university students at the time of study participation. Detailed headache characteristics of the migraine group are summarized in Table 2.

**Table 1.** Demographic Characteristics of the Study Cohort. Abbreviations: BMI (Body Mass Index)

	Migraine		Controls	
Mean age	26.92 (±2.47 years)		26.62 (±3.43 years)	
Sex	12 female, 1 male		12 female, 1 male	
BMI	25.07 ± 3.77 kg/m2		22.81 ± 2.96 kg/m2	
Education	All participants met general university entrance qualifications		All participants met general university entrance qualifications	
Occupation	University Student	61.53%	University Student	61.53%
	Nurse	15.38%	Psychologist	15.38%
	Neuroscientist	7.69%	Lawyer	7.69%
	Architect	7.69%	Physical therapist	7.69%
	HR-manager	7.69%	Not answered	7.69%
	Project manager	7.69%		

1 participant in the migraine group studied while being fulltime employed.

**Table 2.** Headache Characteristics of the Migraine Group as Stated by the Study Participants During the Medical Interview at the Cross-Sectional Appointment. Numbers are Given in Percentage of the Total Number of Headache Patients. Most Frequent Answers are Reported in the Table. Abbreviations: TTH (Tension-type Headache), w (With), w/o (Without), ASA (Acetylsalicylic Acid)

Headache diagnosis	Migraine w aura	Migraine w/o aura	Migraine w aura + TTH	Migraine w/o aura + TTH			
% of study cohort	30.77%	23.07%	15.38%	30.77%			
Mean age at migraine onset for whole study cohort	16.77 ± 6.05 years of age						
Mean time since onset for whole study cohort	10.15 ± 6.88 years						
Accompanying symptoms	Nausea	Neck pain	Phonophobia	Photophobia	Vomiting	Odor sensitivity	
% of study cohort	100%	100%	92.31%	69.23%	30.77%	30.77%	
Headache triggers	Stress	Weather	Menstruation	Changes in sleep rhythm	Sickness	Desk work	Recuperation
% of study cohort	46.15%	38.47%	33.33%	30.77%	23.08%	15.38%	15.38%
Acute medication	Ibuprofen	Triptanes	ASA + Caffeine + Paracetamol	Metamizole	ASA	No acute medication	
% of study cohort	76.92%	46.15%	23.08%	15.38%	7.69%	7.69%	

### Headache and Neck Pain

The average time recorded in the calendars of migraine patients was 83.54 days (99.45%) for headache and 72.23 days (85.99%) for neck pain. In the control group, 75.62 days (90.02%) were recorded on average for both headache and neck pain. In the migraine group, the mean time span from the last headache to the date of the cross-sectional assessment was 4.31 ± 3.17 days for non-migraine headache and 10.77 ± 14.85 days for migraine headache. The mean length of the last headache episode before this assessment was 2.23 ± 1.09 hours.

Regarding headaches, statistically significant differences between groups were reported for headache frequency (*P* = 0.001), headache intensity (*P* = 0.033), headache duration (*P* = 0.008), and frequency of medication intake (*P* = 0.003), with migraine patients scoring higher for all those items than controls (Table 3). Regarding neck pain, migraine patients had a significantly higher neck pain frequency (*P* = 0.023), and duration (*P* = 0.010) compared to controls. There was no significant difference in neck pain intensity (*P* = 0.779) (Table 3).

**Table 3.** Comparison of Headache and Neck Pain Characteristics Between Migraine Patients and Healthy Controls. * Marks Statistical Significance With *P* < 0.05. Abbreviations: NRS (Numerical Rating Scale), N/A (Not Applicable), SD (Standard Deviation), IQR (Interquartile Range), Min (Minimum), Max (Maximum)

	Migraine						Controls						Test values	
	Mean	SD	Median	IQR	Min	Max	Mean	SD	Median	IQR	Min	Max	*Z/t*	*P*
Headache frequency in days/month	8.03	3.11	7.33	3.50	4.67	16.00	1.72	1.62	1.33	1.50	0.00	5.33	*Z* = 3.18	0.001*
Headache intensity on a 10-point NRS	4.83	1.14	4.56	1.81	3.48	7.05	3.87	0.95	3.50	2.00	2.93	5.42	*Z* = 2.13	0.033*
Headache duration in hours	9.26	4.10	7.41	8.47	4.31	15.15	4.33	2.56	3.14	2.44	2.00	10.00	*Z* = 2.67	0.008*
Migraine frequency in days/month	4.74	2.22	5.67	3.50	0.67	8.00	N/A	N/A	N/A	N/A	N/A	N/A	N/A	N/A
Migraine intensity on a 10-point NRS	5.61	1.33	6.00	2.72	3.55	7.19	N/A	N/A	N/A	N/A	N/A	N/A	N/A	N/A
Migraine duration in hours	10.16	4.32	8.00	8.77	5.27	16.35	N/A	N/A	N/A	N/A	N/A	N/A	N/A	N/A
Medication frequency in days/month	4.08	2.81	4.33	4.67	0.00	9.00	0.32	0.52	0.00	0.59	0.00	1.67	*Z* = 2.98	0.003*
Neck pain frequency in days/month	8.35	7.18	7.17	5.21	2.67	28.00	4.01	7.82	1.33	3.33	0.00	28.00	*Z* = 2.28	0.023*
Neck pain intensity on a 10-point NRS	4.31	0.79	4.08	1.49	3.38	5.50	4.08	1.90	3.62	2.60	1.80	8.00	*t* = 0.29	0.779
Neck pain duration in hours	12.73	7.90	12.77	15.51	2.18	24.00	5.19	3.28	4.30	5.93	1.00	10.25	*t* = 3.70	0.010*

### Associations of Neck Pain and Headache over Time

The daily data regarding headache and neck pain are visualized in Figure 3. Regarding OR, it was found that on days with neck pain, it was 5.64 (95% CI [4.14;7.69]) times more likely that an individual reported having a headache on the same day. When considering only migraine attacks, an even higher OR of 7.21 (95% CI [4.95;10.49]) was registered, meaning that it was 7.21 times more likely to have a migraine attack on days with perceived neck pain. When considering only non-migraine headaches - that in the case of this study are equivalent to tension type headaches - the chance was lower with an OR of 1.99 (95% CI [1.33;2.99]), meaning that it was still twice as likely to have a non-migraine headache on days with neck pain. All these differences were statistically significant with *P* < 0.001. In addition, we tested for differences in headache intensity and headache duration in migraine patients on days with and without neck pain (Table 4). We observed that headache intensity and duration were significantly higher on days with neck pain than on days without neck pain (*P* = 0.027, *P* < 0.001).

**Table 4.** Comparison of Migraine Patients’ Headache Intensity and Duration on Days With and Without Neck Pain; Odds Ratio of Different Headache Events Occurring on a Neck-Pain vs Non-neck Pain Day * Marks Statistical Significance With *P* < 0.05. Abbreviations: NRS (Numerical Rating Scale), SD (Standard Deviation), IQR (Interquartile Range), Min (Minimum), Max (Maximum) 95%CI (95% Confidence Interval)

	On neck pain day						On non-neck pain day						Test values	
	Mean	SD	Median	IQR	Min	Max	Mean	SD	Median	IQR	Min	Max	Z	*P*
Headache intensity on a 0-10 NRS	5.37	1.98	5.00	3.00	1.00	10.00	4.80	2.13	5.00	3.00	1.00	10.00	2.21	0.027*
Headache duration in hours	12.44	7.47	10.00	12.00	1.50	24.00	6.74	4.97	6.00	4.00	0.50	24.00	5.79	<0.001*
Odds-ratio	Headache on a neck-pain day			Migraine on a neck-pain day			Non-migraine headache on a neck-pain day							
	5.64 (95% CI [4.14;7.69])			7.21 (95% CI [4.95;10.49])			1.99 (95% CI [1.33;2.99])							

### Assessment of mTrP

After having previously been diagnosed with one mTrP at the screening appointment, one subject in the migraine group did no longer show mTrP at the time of the cross-sectional assessment. Four subjects in the migraine group had an mTrP on the right side, of which one was classified as active and three as latent. Eight subjects had mTrP in the UTM of both sides, all of which were classified as latent. In the control group, one subject had latent mTrP on both sides and one had a latent mTrP on the left side. To summarize, at the cross-sectional appointment, 92.31% of migraine patients were identified with at least one mTrP in the right UTM, while there was only one subject in the control group (7.69%). In the left UTM, 61.54% of migraine patients and 15.38% of controls exhibited at least one mTrP.

### Pressure Pain Thresholds

**Figure 4.**
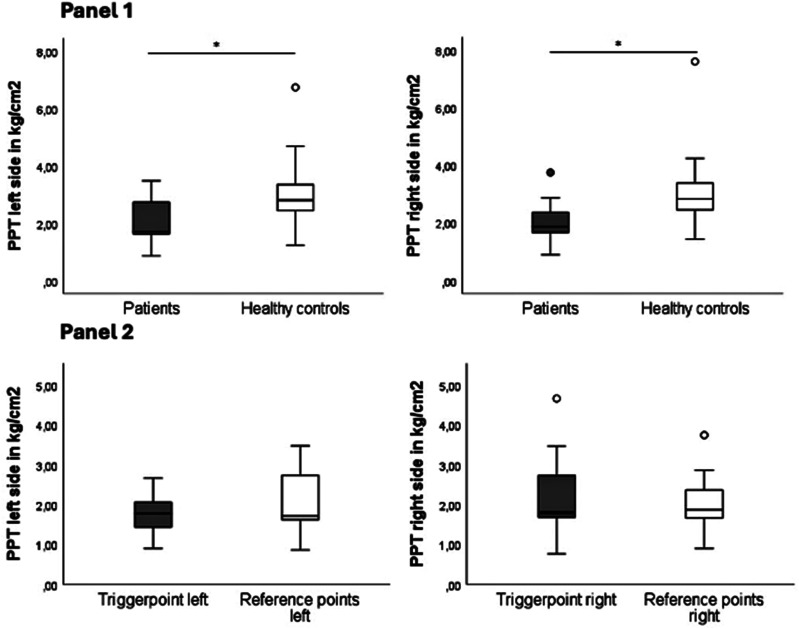
Panel 1 Pressure Pain Thresholds (PPT, in kg/cm^2^) of Patients and Healthy Controls per Body Side (Pooled Values of the Two Reference Points of Each Body Side). Mean Values of Three Rounds of Algometry Were Recorded. * Marks Statistical Significance With *P* < 0.05. Panel 2 Pressure Pain Thresholds (in kg/cm^2^) of Patients Above mTrP Compared to Pooled Values of Reference Points of the UTM of the Same Body Side. Right UTM: 92.31% of Migraine Patients With an mTrP (of which 91.66% Latent, 8.33% Active), One Person in the Control Group (7.69%, Latent). Left UTM: 61.54% of Migraine Patients and 15.38% of Controls Showed an mTrP (all Latent). Abbreviations: PPT (Pressure Pain Thresholds), mTrP (Myofascial Trigger Point), UTM (Upper Trapezius Muscle)

Except for both individual reference points on the left side, migraine patients had significantly lower PPT values than healthy controls, indicating higher pressure sensitivity above the UTM (Table 5, Figure 4, Panel 1). There was no significant difference when comparing the mean PPT values of identified mTrP to the pooled PPT values of reference points of the same side in migraine patients (Figure 4, Panel 2; left: *P* = 0.419, right: *P* = 0.100).

**Table 5.** Pressure Pain Thresholds of Individual Points as Well as Pooled Values for Reference Points of Each Body Side (in kg/cm^2^). Individual Measurements Were Repeated Thrice, and Mean Values Were Calculated. Only One Person in the Control Group Presented With a mTrP on the Right Side. * Marks Statistical Significance With *P* < 0.05. Abbreviations: mTrP (Myofascial Trigger Point), RP (Reference Point), PPT (Pressure Pain Threshold), SD (Standard Deviation), IQR *(*Interquartile Range*)*, Min (Minimum*)*, Max *(*Maximum), N/A (Not Applicable)

	Migraine							Controls							Test values	
	N of points	Mean	SD	Median	IQR	Min	Max	N of points	Mean	SD	Median	IQR	Min	Max	*t/Z*	*P*
PPT pooled RP left	26	2.03	0.76	1.72	1.21	0.87	3.49	26	3.06	1.46	2.80	1.67	1.23	6.73	t = 2.34	0.037*
PPT RP left lateral	13	1.99	0.87	1.80	0.98	0.73	4.00	13	3.01	1.51	2.97	2.23	0.40	6.13	t = 2.14	0.053
PPT RP left medial	13	2.07	0.75	1.78	0.97	1.00	3.43	13	3.12	1.61	2.67	1.37	1.27	7.33	Z = 1.85	0.064
PPT pooled RP right	26	2.03	0.76	1.87	1.06	0.90	3.75	26	3.12	1.58	2.84	1.54	1.44	7.60	Z = 2.48	0.013*
PPT RP right lateral	13	2.06	0.87	1.90	0.86	0.90	4.40	13	3.06	1.54	2.70	1.96	1.20	7.13	Z = 2.38	0.017*
PPT RP right medial	13	2.00	0.73	1.90	1.25	0.90	3.17	13	3.18	1.71	2.70	1.70	1.67	8.07	Z = 2.41	0.016*
PPT mTrP right	12	2.23	1.04	1.80	1.18	0.77	4.67	1	5.10	N/A^a^	N/A^a^	N/A^a^	N/A^a^	N/A^a^	N/A^b^	N/A^b^
PPT mTrP left	8	1.77	0.55	1.79	0.78	0.90	2.67	2	2.49	1.29	2.49	N/A^b^	1.57	3.40	N/A^b^	N/A^b^

^a^No value stated, since n = 1.

^b^No statistical test performed because of low number of mTrP in control group.

### Ultrasound

Significant differences between groups in the thickness of the muscle belly or the fascia at the reference points were observed in 12 out of 24 individual ultrasound parameters (Supplemental Tables 2-4). The difference in muscle thickness between groups was significant for the left lateral reference point in longitudinal (*P* = 0.005) and transversal (*P* < 0.001) direction, as well as the medial reference point on the right transversally (*P* = 0.001) and the left longitudinally (*P* < 0.001). Superior fascial thickness differed significantly for right lateral reference point transversally (*P* = 0.021) and left medial reference point in transversal direction (*P* = 0.034). Inferior fascial thickness was significantly different in both directions for the left lateral reference point (transversal: *P* = 0.004, longitudinal: *P* = 0.002), the left medial reference point (transversal: *P* = 0.044, longitudinal: *P* = 0.041), as well as the right medial reference point (transversal: *P* = 0.040, longitudinal: *P* = 0.046). For all values with significant differences, the control group showed higher numeric values (as normalized on BMI) than migraine patients. After correction for multiple testing, all 4 values for muscle thickness remained significant, with only 2 for inferior fascial thickness (left lateral point in both directions), and none for superior fascial thickness.

In gray scale analysis, neither means, maximum values, nor SD demonstrated a significant difference between migraine patients and healthy controls (Supplemental Tables 5-7). Except for 6 individual measurements, all minimal values were 0.

For migraine intragroup comparison of ultrasound measurements between reference points and mTrP, all measurements of muscle thickness showed significant results (Supplemental Table 8; right UTM longitudinal: *P* = 0.002, transversal: *P* = 0.006, left UTM longitudinal: *P* = 0.002, transversal: *P* = 0.012). While mTrP sites exhibited a thicker muscle belly than reference points on the left UTM, the regions where the reference points at the right side were situated, were thicker than that of mTrP of this side. For fascial thickness, only the right lower UTM fascia in longitudinal direction yielded a significant result (*P* = 0.006). All of these results remained significant after correction.

Gray scale comparison of mTrP and reference points (Supplemental Table 9) resulted in significant results for the right-side UTM in transversal direction for mean (*P* = 0.009), SD (*P* < 0.001) and maximum (*P* = 0.014) values, while only SD remained significant after correction.

## Discussion

The aim of this prospective study was to further explore muscular symptoms as well as myofascial findings in the neck region of patients affected by migraine compared to healthy controls. Migraine patients showed significantly higher neck pain frequency and duration than healthy controls. Our findings are in line with the so far existing literature describing neck pain as a common symptom in migraine patients: neck pain is an even more common symptom during a migraine attack than nausea, with almost twice the frequency during episodes with moderate headache intensity.^20^ In a systematic review, neck pain frequency was reported to be 77% in migraine patients vs 23% in healthy controls.^14^ In a narrative review, an association between subjective neck pain and migraine attacks was observed, and chronic neck pain was associated with a higher rate of migraine attacks and higher mean headache intensity.^15^

Furthermore, our results highlight the timely association of headaches and migraine attacks and neck pain: it was 5.6 times more likely to have a migraine or non-migraine headache on a day with neck pain than on days without neck pain. This was especially pronounced for migraines, which were 7.21 times more likely to occur on neck pain days. It is remarkable that some patients had a complete (n = 3) or nearly complete (n = 2 with >90% overlap) match of neck pain and headache days, meaning that neck pain never or only very rarely occurred without a headache episode on the same day. This is congruent to another study analyzing the temporal relationship of neck pain and migraine episodes.^44^ In this previous study, neck pain and migraine occurrences were grouped into different categories of headache and neck pain relation ranging from “ictal neck pain only” over “frequent ictal and interictal neck pain” and “infrequent interictal and ictal neck pain” to non-decipherable patterns.^44^ In contrast, we focused strictly on the day-by-day overlap of headache and neck pain, while Liang et al relied on a wider categorization of interrelated patterns. Importantly, in our migraine cohort, the presence of myofascial involvement clinically reflected by the presence of at least one mTrP was an inclusion criterion, leading to a preselection of patients with myofascial involvement in the neck area in general. Further, there was no specific subcategorization of patterns in our study due to a relatively small sample size and our primary goal being the comparison to a healthy control group. The pathophysiological model of the TCC as a link between headache and neck pain is well established.^4,45,46^ Nevertheless, the question of cause and consequence remains unanswered so far: it is not yet clear whether or to what extent the repeated input of nociceptive stimuli from the neck musculature is the basis of the emergence of central pain mechanisms, or whether cranio-cervical symptoms are a result of altered central activity in migraine pathophysiology (bottom-up vs top-down hypothesis).

Along with the higher frequency and duration of neck pain, migraine patients had lower PPT above the UTM than healthy controls, translating to higher pressure sensitivity as interpreted as a surrogate of the myofascial peripheral sensitization. These findings are in line with previous investigations reporting lower PPT in migraine patients.^24,25^ Regarding mTrP, approximately 15% of healthy controls were identified with at least one mTrP in the UTM of either side. A cross-sectional study on the prevalence of latent mTrP in the UTM in 220 healthy individuals found an occurrence of 20% and 23% for the right and left side, respectively.^47^ While our study does not allow for a distinct analysis of difference in the presence of active and latent mTrP between the study groups due to the study design, other work concluded that there likely is a significantly higher number of latent as well as active mTrP in migraine patients.^23,48^ Interestingly, when comparing PPT above the mTrP to those above the ipsilateral reference points in migraine patients, no significant difference was detected. This finding may indicate that the higher level of pressure sensitivity affects the whole muscle instead of being only limited to a focal alteration conceptualized as mTrP.

In our cohort, significant differences in relative muscular and fascia thickness between migraine patients and healthy controls were observed in 4 out of 8 ultrasound measurements for muscle thickness, 2 out of 8 for upper-, and 6 out of 8 for lower fascial thickness, while no significant differences in gray scale measurements were found. This may partly be due to the small sample size in general and the low number of healthy controls diagnosed with mTrP, leading to a high intra-group heterogeneity.

Gray scale analysis comparing tissue composition between migraine patients and healthy participants was not focused on mTrP specifically but encompassed a distinct area of the muscle belly surrounding the reference points. Interestingly, although clinical differences exist regarding the PPT, these differences do not reflect in overall tissue composition as assessed by gray scaling. It has been demonstrated by other researchers that patients suffering from myofascial pain syndrome demonstrate significant differences in echogenicity of the trapezius muscles.^49^ Additionally, MRI based T2 mapping has demonstrated detectable tissue alterations within the UTM in headache patients. Thus, more research in this direction would be valuable in the migraine cohort.^30-32^

Within the migraine group some differences within the region of mTrP and the reference points were detectable in this study – an observation that could be helpful in better characterizing the individual myofascial involvement of different patients. Future studies may also benefit from more sophisticated methods of analysis, even including machine learning algorithms which may detect differences we were not able to observe or from the inclusion of other techniques, eg, shear wave elastography.

Regarding fascia thickness, an intraoperative study found that 94% of patients undergoing greater occipital nerve decompression surgery for occipital neuralgia, headache or migraine exhibited thickening of the trapezius fascia with signs of inflammation.^50^ A thickening of the muscle fascia for the sternocleidomastoid muscle and the scalenus medius muscle on the right side, as diagnosed by ultrasound, was reported in patients with chronic neck pain.^51^ We were not able to reproduce these findings for our migraine cohort by B-mode ultrasound.

Comparison of ultrasound values between mTrP and reference points of same-side UTM in migraine patients showed significant results for muscle thickness quotients of both sides, indicating a difference in relative muscle thickness between mTrP and the rest of the UTM. However, recorded differences did not point in the same direction, as measurements for mTrP showed higher muscle thickness on the left, but lower thickness on the right side. Which inferences may be drawn from this is unclear as, again, this must be seen in the context of a relatively small sample size. Additionally, confounding factors like position of mTrP within the trapezius muscle, which is not uniformly thick, may influence measurements. Again, changes in the musculature’s structure, as well as its fascia, may not be restricted to focal points characterized as mTrP. If indeed neurogenic inflammatory processes are a muscle-wide phenomenon, comparisons of larger sample sizes of affected and non-affected UTM may show more indicatory results.

Despite not being able to produce a reliable differentiation of measures within the UTM of migraine patients and controls, possible applications of ultrasound as a point-of-care modality in the context of migraine care remain an area of high interest and should therefore be investigated in a larger-scaled approach. B-mode sonography including gray scaling may still prove to be useful as a point of care tool regarding the diagnosis of a myofascial involvement in the case of primary headache disorders and in monitoring its trajectory. Presently, we would like to emphasize the potential in identifying those patients that may benefit from muscular-centered treatments within the tailored multimodal approach and to objectively and quantitatively monitor their effects on behalf of ultrasound measures and corresponding manual examinations.

The herein presented results point to several clinical implications. Health care providers should be aware of the possible myofascial involvement when diagnosing patients or assessing those already diagnosed with migraine. Even though muscular symptoms may not be present in all patients, data show that patients affected by any aspect of muscular involvement are not the exception.^14^ In this context, the possible pathophysiological link between migraine and the myofascial component should be kept in mind. Myofascial peripheral sensitization leads to a higher rate of generation of nociceptive information, which is in turn transmitted via the TCC to the pain-processing system and integrated herein.^6-9^ If migraine patients have been identified with muscular symptoms, a multi-modal treatment approach including methods targeting the involved muscles should be offered.^28,52-56^ The aim of muscular-focused treatment is preventive in the means of achieving a decrease in headache frequency and/or a decrease in the burden every single headache attack comes along with, alongside the beneficial effects locally on the musculature. Thus, an important goal of multimodal headache care could be tackled. Treatments targeting the muscles offer patients an individualized approach of addressing the symptoms directly where they experience them. This easily comprehensible concept gives patients a sense of self-empowerment that stands in contrast to a common routine of feeling at the mercy of their disease. This is especially pronounced in self-directed treatment options. Therapeutic strategies that focus on the association of headache and myofascial symptoms in migraine include dry needling,^57^ physiotherapy including manual therapy as well as self-directed exercises,^58^ and progressive muscle relaxation (PMR).^59^ Furthermore, non-invasive peripheral neurostimulation techniques like repetitive neuromuscular magnetic stimulation (rNMS) use the bottom-up approach to modulate not only muscular but also headache symptoms of migraine.^60-66^ In this regard, rNMS includes the patient-centric approach of offering treated patients the ability to self-direct position and intensity of stimulation.^67^ While the association over time between neck pain and headache in this study is of potential pathophysiological and clinical interest, its immediate relevance is not entirely clear yet.

A strength of the presented analyses is that the healthy control group was thoroughly matched to the migraine group in age, sex, BMI, and demographics like educational level and employment. Moreover, strict inclusion criteria limited the presence of possibly confounding neurologic, psychiatric, or musculoskeletal conditions. All subjects were asked to update their headache and neck pain calendars on a daily basis to minimize any recall biases, which is also reflected in the high adherence to the calendars of 91.37%. Participants were prospectively followed over a 3-month period without any headache-related intervention or change in their daily routine. By assessing headache and neck pain calendars simultaneously, headache and neck pain were not put in direct connection to one another beforehand, minimizing any biases in the self-assessment of headache and neck pain and allowing an independent analysis.

Limitations of this study include the high percentage of females in the migraine group, which does not accurately represent the real-life distribution across the general population of migraine patients.^2^ However, in the examined age group (18-35 years), there is a higher prevalence of migraine in women than men.^68^ Further, in this study cohort, a relevant proportion of patients were affected by migraine plus tension type headache often conceptualized as “mixed-type” headache. This coexistence is frequently diagnosed in particular with raising headache frequency. Given this circumstance some researchers discuss both headache types more as the 2 ends of one spectrum than as 2 different entities.^16,69^ Thus, the compilation of the study cohort reflects a real-world sample of patients and may underline the thorough headache assessment and classification the patients undergo during the screening and inclusion process of the study.

Moreover, the sample size was rather small with 13 migraine patients and 13 healthy controls. The limited sample size is partly due to the personnel- and time-consuming nature of the study’s setup, and partly to the thorough matching of migraine and healthy participants. Hence, based on the insights reported here, the comprehensive approach of this study should be further developed and followed up by larger-scaled studies. Thereby, given a larger sample size, it will be important to analyze subgroups according to different headache types, ie, migraine, TTH, migraine plus TTH, and post-traumatic headache (PTH) and to stratify according to headache frequency, ie, episodic, high-frequency episodic, and chronic. Furthermore, it should be noted that the presence of mTrP was an inclusion criterion in the migraine group but not in the control group. Hence, no conclusions on the distribution of neck pain or muscular involvement in the general migraine population can be drawn from this study.

Another limitation are examiner-dependent factors impacting on intra- and interrater reliability. We aimed to minimize bias by standardizing the measurements of algometry and ultrasound as much as possible. This included the placement of reference points in relation to anatomic landmarks, structured instructions for the positioning of the ultrasound probe, and a strict sequence to follow for the individual measurements. Furthermore, we included a repetition of measurements for each individual point to correct for a certain variance in measurements.

As has been shown in this work, the muscular component plays a role in migraine; however, the extent of its influence is largely unknown, as are the specific subgroups of patients that may profit from more focused diagnosis and therapy. Examinations by a physical therapist were largely limited to the detection of active and latent mTrP, the reason being limited availability of experienced professionals. Since presence of an mTrP was the only indicator of myofascial involvement in our patients and controls, we cannot make any statement on other musculoskeletal components. Myofascial involvement contains much more than only mTrP, thus future studies should include a wider range of patients with more diverse myofascial symptoms. Besides more extensive screening by physical therapists, other tools, such as self-rating-assessments like the Neck Disability Index (NDI)^70,71^ may be helpful for identifying patients with muscular symptoms and alterations. Though migraine is one of the most relevant headache disorders, it is not the only one where such myofascial involvement may play a role.

## Conclusion

This study highlights the importance of muscular symptoms and myofascial findings in the UTM in migraine patients compared to healthy controls. In this cohort of migraine patients with diagnosed mTrP in the UTM, neck pain was not only more common and lasted longer than in healthy controls, it was also highly temporally associated with the occurrence of headache, especially episodes of migraine. General sensitivity of the UTM, modeled with PPT, was higher in patients than controls. Health care providers should be aware of the frequent co-occurrence of symptoms, as it may lead to both better diagnoses, as well as tailored treatment approaches. Though individual significant differences were demonstrated, B-mode ultrasound did not show consistent differences between groups and will need additional research and possibly different technical approaches to show whether it may prove to be a viable, objective addition to the clinical diagnostic process.

## Supplemental Material

Supplemental Material - The Temporal Associations of Neck Pain and Headache – Implications for the Diagnostic Approach to the Myofascial Involvement in Migraine Supplemental Material for The Temporal Associations of Neck Pain and Headache – Implications for the Diagnostic Approach to the Myofascial Involvement in Migraine by Corinna Börner-Schröder, Thomas Lachhammer, Paula Behrendt, Theresa Pfeifer, Paulina Kolorz, Sarah Lense, Julie Pompignoli, Miriam Reichert, Severin Schramm, Florian Heinen, Nico Sollmann, Michaela Bonfert in Journal of Central Nervous System Disease

## Data Availability

The data presented in this study are available on request from the corresponding author. The data are not publicly available due to the sensitive character of clinical data.*

## Appendix

### Abbreviations

95% CI: 95% confidence interval
ASA: acetylsalicylic acid
B-mode: brightness mode
BMI: body mass index
DMKG: Deutsche Migräne-und Kopfschmerzgesellschaft [German Migraine and Headache Society]
DRKS: Deutsches Register Klinischer Studien [German Clinical Trials Registry]
ICHD-3: International Classification of Headache Disorders third edition
IQR: interquartile range
Max: maximum
Min: minimum
MRI: magnetic resonance imaging
mTrP: myofascial trigger point
N/A: not applicable
NDI: neck disability index
NRS: numerical rating scale
OR: odds ratio
PMR: progressive muscle relaxation
PPT: pressure pain threshold
PTH: post-traumatic headache
rNMS: repetitive neuromuscular magnetic stimulation
ROI: region of interest
SD: standard deviation
TCC: trigemino-cervical complex
TTH: tension-type headache
UTM: upper trapezius muscle
w: with
w/o: without
