# Supplementary material for: The Temporal Associations of Neck Pain and Headache – Implications for the Diagnostic Approach to the Myofascial Involvement in Migraine
Source: J Cent Nerv Syst Dis. 2025 Dec 11;17:11795735251404279. doi: 10.1177/11795735251404279 (PMC12699004; doi:10.1177/11795735251404279)
Supplement: Supplemental Material - The Temporal Associations of Neck Pain and Headache – Implications for the Diagnostic Approach to the Myofascial Involvement in Migraine [file sj-pdf-1-cns-10.1177_11795735251404279.pdf]

Supplement Table 1 Outcome measures. Measure, source material used, participants included, as well as corresponding statistical tests. Abbreviations: PPT (pressure pain threshold), mTrP (myofascial trigger point), UTM (upper trapezius muscles), SD (standard deviation).

Measure	Method	Participants	Statistical test	Time period recorded
Frequency, intensity, and duration of headache	Headache calendars	Patients (n=13) and controls (n=13)	Wilcoxon test, t-test for paired samples	84 days (longitudinal)
Frequency, intensity, and duration of neck pain	Neck pain calendars	Patients (n=12) and controls (n=12)	Wilcoxon test, t-test for paired samples	84 days (longitudinal)
Frequency of intake of analgesic medication due to headache	Headache and neck pain calendars	Patients (n=13) and controls (n=13)	Wilcoxon test	84 days (longitudinal)
Association of neck pain and migraine days (over time)	Headache and neck pain calendars	Patients only (939 individual days)	Chi-squared test, Odds ratio	84 days (longitudinal)
Association of neck pain and non-migraine headache days (over time)	Headache and neck pain calendars	Patients only (939 individual days)	Chi-squared test, Odds ratio	84 days (longitudinal)
Headache intensity and duration on neck pain/non-neck pain days	Headache and neck pain calendars	Patients only (939 individual days)	Wilcoxon test	84 days (longitudinal)
PPT above the UTM at all individual reference points, subjectively most painful mTrP on each body side, and reference points pooled for each body side	PPT measurement by algometer	Patients (n=13) and controls (n=13)	Wilcoxon test, t-test for paired samples	Once (cross-sectional)
PPT above the UTM at mTrP vs. pooled PPT of reference points on the same body side	PPT measurement by algometer	Patients only (n=12 on the right, n=8 on the left)	T-test for paired samples	Once (cross-sectional)
Thickness of muscle belly, upper and lower fascia of the UTM	Muscular ultrasound	Patients (n=12) and controls (n=12)	Wilcoxon test, t-test for paired samples	Once (cross-sectional)
Gray scale values of the UTM	Muscular ultrasound	Patients (n=11) and controls (n=11)	Wilcoxon test, t-test for paired samples	Once (cross-sectional)
Pooled relative thickness of reference point muscle belly, upper and lower fascia thickness vs. mTrP values of the same body side	Muscular ultrasound	Patients only (n=12 on the right, n=8 on the left)	Wilcoxon test, t-test for paired samples	Once (cross-sectional)
Pooled gray scale values (mean, SD and maximum) of reference points vs. mTrP of the same body side	Muscular ultrasound	Patients only (n=11 on the right, n=8 on the left)	Wilcoxon test, t-test for paired samples	Once (cross-sectional)

Supplement Table 2. Comparison of muscle thickness/BMI-ratio in mm/kg/m2. * marks statistical significance with p<0.05, † marks statistical significance after correction for multiple testing. Abbreviations: SD standard deviation, IQR interquartile range, Min minimum, Max maximum, UTM upper trapezius muscle, mTrP myofascial trigger point, N/A not applicable.

**UTM – muscle belly**	**Migraine**						**Controls**						**Test values**	
	Mean	SD	Median	IQR	Min	Max	Mean	SD	Median	IQR	Min	Max	*t/Z*	*p*
**Lateral reference point**														
Right longitudinal	0.326	0.075	0.325	0.120	0.190	0.450	0.369	0.107	0.364	0.110	0.210	0.640	*Z*=1.32	0.187
Right transversal	0.363	0.076	0.347	0.129	0.250	0.530	0.389	0.091	0.372	0.130	0.270	0.640	*Z*=1.21	0.226
Left longitudinal	0.298	0.107	0.280	0.090	0.160	0.580	0.358	0.106	0.306	0.150	0.250	0.670	*Z*=2.80	0.005*†
Left transversal	0.331	0.080	0.334	0.120	0.190	0.490	0.399	0.079	0.386	0.100	0.300	0.610	*Z*=3.33	<0.001*†
**Medial reference point**														
Right longitudinal	0.273	0.052	0.264	0.070	0.200	0.400	0.320	0.121	0.316	0.200	0.150	0.590	*Z*=1.90	0.057
Right transversal	0.341	0.049	0.344	0.080	0.260	0.450	0.377	0.099	0.377	0.160	0.220	0.560	*t*=3.59	0.001*†
Left longitudinal	0.217	0.069	0.208	0.070	0.100	0.390	0.343	0.060	0.341	0.080	0.240	0.460	*t*=8.57	<0.001*†
Left transversal	0.274	0.062	0.272	0.070	0.170	0.410	0.307	0.090	0.303	0.110	0.140	0.530	*t*=1.58	0.125
**mTrP**														
Right longitudinal	0.254	0.053	0.261	0.040	0.150	0.360	0.136	0.014	0.136	N/A[1]	0.120	0.150	N/A[2]	N/A[2]
Right transversal	0.294	0.054	0.309	0.090	0.210	0.430	0.291	0.022	0.302	N/A[1]	0.270	0.310	N/A[2]	N/A[2]
Left longitudinal	0.261	0.042	0.266	0.050	0.160	0.330	0.260	0.024	0.262	N/A[1]	0.230	0.280	N/A[2]	N/A[2]
Left transversal	0.301	0.042	0.305	0.070	0.230	0.370	0.227	0.007	0.230	N/A[1]	0.220	0.230	N/A[2]	N/A[2]

[1]Low number of data points with sufficient image quality
[2]No statistical test performed because of low number of mTrP in control group

Supplement Table 3. Comparison of upper fascia thickness/BMI-ratio in mm/kg/m2. * marks statistical significance with p<0.05, † marks statistical significance after correction for multiple testing. Abbreviations: SD standard deviation, IQR interquartile range, Min minimum, Max maximum, UTM upper trapezius muscle, mTrP myofascial trigger point, N/A not applicable.

**UTM – superior fascia**	**Migraine**						**Controls**						**Test values**	
	Mean	SD	Median	IQR	Min	Max	Mean	SD	Median	IQR	Min	Max	*t/Z*	*p*
**Lateral reference point**														
Right longitudinal	0.034	0.011	0.320	0.010	0.020	0.060	0.035	0.008	0.034	0.010	0.020	0.050	*t*=0.50	0.960
Right transversal	0.030	0.005	0.028	0.010	0.020	0.040	0.034	0.008	0.032	0.010	0.020	0.060	*Z*=2.31	0.021*
Left longitudinal	0.031	0.008	0.031	0.010	0.020	0.050	0.033	0.008	0.032	0.010	0.020	0.050	*Z*=1.46	0.144
Left transversal	0.035	0.013	0.032	0.020	0.020	0.070	0.035	0.008	0.035	0.010	0.020	0.050	*Z*=0.33	0.741
**Medial reference point**														
Right longitudinal	0.040	0.013	0.039	0.010	0.020	0.080	0.037	0.008	0.037	0.010	0.020	0.050	*Z*=0.23	0.820
Right transversal	0.035	0.012	0.031	0.010	0.020	0.060	0.037	0.008	0.037	0.010	0.020	0.060	*t*=1.34	0.190
Left longitudinal	0.043	0.034	0.033	0.010	0.020	0.210	0.038	0.009	0.037	0.010	0.020	0.060	*Z*=1.05	0.295
Left transversal	0.033	0.014	0.029	0.010	0.020	0.080	0.038	0.009	0.037	0.010	0.020	0.060	*Z*=2.12	0.034*
**mTrP**														
Right longitudinal	0.031	0.007	0.031	0.010	0.020	0.040	0.035	0.001	0.035	N/A[1]	0.030	0.040	N/A[2]	N/A[2]
Right transversal	0.030	0.006	0.030	0.010	0.020	0.040	0.032	0.006	0.033	N/A[1]	0.030	0.040	N/A[2]	N/A[2]
Left longitudinal	0.032	0.010	0.029	0.020	0.020	0.060	0.024	0.001	0.025	N/A[1]	0.020	0.030	N/A[2]	N/A[2]
Left transversal	0.032	0.009	0.031	0.020	0.020	0.050	0.028	0.005	0.025	N/A[1]	0.020	0.030	N/A[2]	N/A[2]

[1]Low number of data points with sufficient image quality
[2]No statistical test performed because of low number of mTrP in control group

Supplement Table 4. Comparison of lower fascia thickness/BMI-ratio in mm/kg/m2. * marks statistical significance with p<0.05, † marks statistical significance after correction for multiple testing. Abbreviations: SD standard deviation, IQR interquartile range, Min minimum, Max maximum, UTM upper trapezius muscle, mTrP myofascial trigger point, N/A not applicable.

**UTM – inferior fascia**	**Migraine**						**Controls**						**Test values**	
	Mean	SD	Median	IQR	Min	Max	Mean	SD	Median	IQR	Min	Max	*t/Z*	*p*
**Lateral reference point**														
Right longitudinal	0.030	0.008	0.029	0.010	0.010	0.050	0.032	0.007	0.033	0.010	0.020	0.050	*t*=1.26	0.216
Right transversal	0.030	0.005	0.029	0.010	0.010	0.040	0.032	0.008	0.030	0.010	0.020	0.050	*t*=1.54	0.134
Left longitudinal	0.029	0.006	0.028	0.010	0.020	0.040	0.035	0.008	0.035	0.010	0.020	0.050	*Z*=3.13	0.002*†
Left transversal	0.029	0.006	0.028	0.010	0.020	0.040	0.034	0.007	0.033	0.010	0.020	0.050	*t*=3.15	0.004*†
**Medial reference point**														
Right longitudinal	0.028	0.006	0.028	0.010	0.020	0.040	0.032	0.008	0.032	0.010	0.020	0.050	*t*=2.10	0.046*
Right transversal	0.028	0.006	0.029	0.010	0.010	0.040	0.031	0.006	0.030	0.010	0.020	0.040	*t*=2.16	0.040*
Left longitudinal	0.029	0.009	0.028	0.010	0.020	0.050	0.031	0.006	0.031	0.010	0.020	0.040	*Z*=2.04	0.041*
Left transversal	0.027	0.005	0.027	0.010	0.020	0.040	0.030	0.008	0.030	0.010	0.020	0.050	*t*=2.10	0.044*
**mTrP**														
Right longitudinal	0.027	0.005	0.026	0.010	0.020	0.040	0.034	0.008	0.034	N/A[1]	0.030	0.040	N/A[2]	N/A[2]
Right transversal	0.029	0.006	0.027	0.010	0.020	0.040	0.034	0.001	0.033	N/A[1]	0.030	0.040	N/A[2]	N/A[2]
Left longitudinal	0.026	0.007	0.028	0.010	0.010	0.040	0.030	0.002	0.030	N/A[1]	0.030	0.030	N/A[2]	N/A[2]
Left transversal	0.027	0.005	0.026	0.010	0.020	0.040	0.030	0.003	0.032	N/A[1]	0.030	0.030	N/A[2]	N/A[2]

[1]Low number of data points with sufficient image quality
[2]No statistical test performed because of low number of mTrP in control group

Supplement Table 5. Mean UTM gray scale values of the innermost 75% of muscular tissue surrounding a reference point/mTrP. Gray scale values are given on a numeric scale from 0-255. * marks statistical significance with p<0.05, † marks statistical significance after correction for multiple testing. Abbreviations: SD standard deviation, IQR interquartile range, Min minimum, Max maximum, UTM upper trapezius muscle, mTrP myofascial trigger point, N/A not applicable.

**UTM – Gray Scale**	**Migraine**						**Controls**						**Test values**	
	Mean	SD	Median	IQR	Min	Max	Mean	SD	Median	IQR	Min	Max	*t/Z*	*p*
**Lateral reference point**														
Right longitudinal	48.46	7.91	46.83	14.29	37.65	62.66	40.60	12.27	43.52	25.27	19.91	56.59	*t*=1.58	0.149
Right transversal	51.80	9.95	52.52	11.40	31.16	65.55	43.83	8.33	45.89	11.92	29.96	58.65	*t*=2.04	0.068
Left longitudinal	45.40	11.59	46.80	22.53	29.13	60.18	40.65	13.91	43.37	14.06	18.95	62.86	*t*=1.92	0.091
Left transversal	46.79	13.67	49.41	17.70	18.74	67.80	42.95	11.98	40.90	18.95	17.49	57.02	*t*=0.79	0.446
**Medial reference point**														
Right longitudinal	40.96	12.04	38.87	12.07	16.71	59.33	36.52	14.11	42.73	22.00	10.64	54.48	*t*=0.44	0.670
Right transversal	39.49	10.79	39.49	18.23	19.66	56.10	37.05	11.65	42.40	15.14	12.49	48.26	*t*=0.01	0.996
Left longitudinal	38.46	11.94	39.50	12.29	14.54	52.15	35.89	12.44	36.94	14.40	14.15	58.80	*t*=1.22	0.251
Left transversal	41.93	12.08	41.74	14.80	19.01	57.91	37.58	13.45	38.07	25.21	18.08	59.88	*t*=1.32	0.215
**mTrP**														
Right longitudinal	36.82	11.73	35.46	20.54	18.03	53.92	39.47	N/A[1]	N/A[1]	N/A[1]	N/A[1]	N/A[1]	N/A[2]	N/A[2]
Right transversal	33.89	11.79	33.07	10.53	14.84	59.32	34.44	N/A[1]	N/A[1]	N/A[1]	N/A[1]	N/A[1]	N/A[2]	N/A[2]
Left longitudinal	42.31	10.27	42.04	20.58	29.46	55.92	N/A[1]	N/A[1]	N/A[1]	N/A[1]	N/A[1]	N/A[1]	N/A[2]	N/A[2]
Left transversal	45.18	11.38	44.23	22.81	32.46	60.90	N/A[1]	N/A[1]	N/A[1]	N/A[1]	N/A[1]	N/A[1]	N/A[2]	N/A[2]

[1]Low number of data points with sufficient image quality

[2]No statistical test performed because of low number of mTrP in control group

Supplement Table 6. Standard deviation of UTM gray scale values of the innermost 75% of muscular tissue surrounding a reference point/mTrP. Gray scale values are given on a numeric scale from 0-255. * marks statistical significance with p<0.05, † marks statistical significance after correction for multiple testing. Abbreviations: SD standard deviation, IQR interquartile range, Min minimum, Max maximum, UTM upper trapezius muscle, mTrP myofascial trigger point, N/A not applicable.

**UTM – Gray scale**	**Migraine**						**Controls**						**Test values**	
	Mean	SD	Median	IQR	Min	Max	Mean	SD	Median	IQR	Min	Max	*t/Z*	*p*
**Lateral reference point**														
Right longitudinal	24.59	3.03	24.56	3.24	20.01	32.01	23.31	2.37	23.25	3.26	18.62	26.92	*t*=1.52	0.161
Right transversal	27.64	3.82	28.98	6.02	21.47	34.52	25.21	2.88	26.21	4.88	21.44	30.22	*t*=1.73	0.114
Left longitudinal	25.17	2.85	23.79	4.97	22.63	30.16	23.37	3.76	24.17	4.04	15.63	29.60	*Z*=0.89	0.374
Left transversal	27.08	5.17	27.65	6.42	14.17	32.76	25.24	3.80	23.22	3.84	21.20	34.17	*Z*=0.98	0.328
**Medial reference point**														
Right longitudinal	22.86	5.73	21.09	8.23	15.83	34.11	22.27	3.07	23.21	3.92	16.60	27.30	*t*=0.10	0.993
Right transversal	24.77	4.97	23.69	7.17	18.59	33.34	23.83	2.86	24.41	2.55	17.49	27.27	*t*=0.17	0.867
Left longitudinal	21.30	4.55	20.73	6.11	14.06	29.34	21.39	2.43	21.85	4.03	16.49	24.80	*t*=0.06	0.952
Left transversal	24.56	4.43	25.83	5.88	16.97	31.61	24.05	3.22	23.83	6.80	19.95	28.66	*t*=0.33	0.747
**mTrP**														
Right longitudinal	20.22	4.70	19.76	7.00	13.73	29.76	20.72	N/A[1]	N/A[1]	N/A[1]	N/A[1]	N/A[1]	N/A[2]	N/A[2]
Right transversal	20.49	4.59	19.54	5.16	14.69	31.22	17.58	N/A[1]	N/A[1]	N/A[1]	N/A[1]	N/A[1]	N/A[2]	N/A[2]
Left longitudinal	23.09	5.44	22.39	8.70	15.10	31.89	N/A[1]	N/A[1]	N/A[1]	N/A[1]	N/A[1]	N/A[1]	N/A[2]	N/A[2]
Left transversal	26.34	3.95	28.57	6.03	19.70	30.03	N/A[1]	N/A[1]	N/A[1]	N/A[1]	N/A[1]	N/A[1]	N/A[2]	N/A[2]

[1]Low number of data points with sufficient image quality

[2]No statistical test performed because of low number of mTrP in control group

Supplement Table 7. Maximum of UTM gray scale values of the innermost 75% of muscular tissue surrounding a reference point/mTrP. Gray scale values are given on a numeric scale from 0-255. * marks statistical significance with p<0.05, † marks statistical significance after correction for multiple testing. Abbreviations: SD standard deviation, IQR interquartile range, Min minimum, Max maximum, UTM upper trapezius muscle, mTrP myofascial trigger point, N/A not applicable.

**UTM – Gray scale**	**Migraine**						**Controls**						**Test values**	
	Mean	SD	Median	IQR	Min	Max	Mean	SD	Median	IQR	Min	Max	*t/Z*	*p*
**Lateral reference point**														
Right longitudinal	186.55	13.52	183.33	20.00	160.33	207.00	179.45	14.24	180.00	27.67	158.00	200.67	*t*=1.25	0.242
Right transversal	207.76	21.92	212.00	32.00	165.67	236.67	196.73	17.50	197.67	19.33	166.00	220.67	*t*=1.47	0.173
Left longitudinal	178.67	24.84	183.33	38.33	134.33	203.00	171.12	23.10	173.33	30.67	128.33	203.67	*t*=0.92	0.384
Left transversal	194.29	33.47	204.33	26.67	104.67	224.00	191.24	18.71	196.67	38.00	164.33	219.00	*Z*=1.02	0.306
**Medial reference point**														
Right longitudinal	157.63	34.23	152.33	60.50	120.33	213.33	147.88	28.63	149.33	51.67	104.33	189.33	*t*=0.42	0.685
Right transversal	176.03	28.19	169.50	55.25	138.00	218.67	171.33	21.93	171.67	24.67	128.67	209.00	*t*=0.44	0.966
Left longitudinal	146.80	29.76	150.33	53.83	108.00	198.33	142.52	15.40	139.67	17.67	115.00	170.33	*t*=0.63	0.544
Left transversal	179.94	26.55	180.67	33.00	124.33	214.33	168.70	26.80	177.67	41.67	116.00	206.67	*t*=1.24	0.245
**mTrP**														
Right longitudinal	149.67	36.54	153.67	58.08	102.33	216.00	140.33	N/A[1]	N/A[1]	N/A[1]	N/A[1]	N/A[1]	N/A[2]	N/A[2]
Right transversal	160.07	32.65	157.33	45.25	113.00	221.67	135.67	N/A[1]	N/A[1]	N/A[1]	N/A[1]	N/A[1]	N/A[2]	N/A[2]
Left longitudinal	167.79	37.05	178.50	66.92	107.33	212.33	N/A[1]	N/A[1]	N/A[1]	N/A[1]	N/A[1]	N/A[1]	N/A[2]	N/A[2]
Left transversal	187.76	25.21	169.67	46.00	146.33	212.33	N/A[1]	N/A[1]	N/A[1]	N/A[1]	N/A[1]	N/A[1]	N/A[2]	N/A[2]

[1]Low number of data points with sufficient image quality

[2]No statistical test performed because of low number of mTrP in control group

Supplement Table 8. Comparison of migraine patients' muscle/fascial thickness to BMI-ratio in mm/kg/m^2 of pooled reference points to mTrP of the same side. * marks statistical significance with p<0.05, † marks statistical significance after correction for multiple testing. Abbreviations: SD standard deviation, IQR interquartile range, Min minimum, Max maximum, UTM upper trapezius muscle, mTrP myofascial trigger point, RP reference points.

	**RP**						**mTrP**						**Test values**	
	Mean	SD	Median	IQR	Min	Max	Mean	SD	Median	IQR	Min	Max	*t/Z*	p
**Muscle thickness**														
right UTM longitudinal	0.306	0.056	0.310	0.087	0.204	0.409	0.254	0.053	0.261	0.044	0.153	0.356	Z=3.01	0.002*†
right UTM transversal	0.352	0.059	0.340	0.100	0.269	0.474	0.294	0.054	0.309	0.092	0.205	0.431	Z=2.69	0.006*†
left UTM longitudinal	0.230	0.048	0.221	0.040	0.151	0.346	0.261	0.042	0.266	0.055	0.162	0.333	Z=2.93	0.002*†
left UTM transversal	0.279	0.053	0.270	0.086	0.205	0.376	0.300	0.042	0.305	0.071	0.227	0.375	t=2.18	0.012*†
**Upper fascial thickness**														
right UTM longitudinal	0.036	0.008	0.036	0.009	0.020	0.054	0.031	0.007	0.031	0.012	0.019	0.044	t=3.00	0.006*†
right UTM transversal	0.030	0.005	0.031	0.009	0.021	0.039	0.030	0.006	0.030	0.006	0.017	0.042	t=0.01	0.989
left UTM longitudinal	0.037	0.020	0.031	0.013	0.021	0.120	0.032	0.010	0.028	0.016	0.020	0.062	Z=0.95	0.353
left UTM transversal	0.035	0.015	0.035	0.014	0.019	0.070	0.033	0.009	0.031	0.015	0.015	0.046	Z=0.49	0.643
**Lower fascial thickness**														
right UTM longitudinal	0.029	0.004	0.029	0.006	0.020	0.036	0.027	0.005	0.026	0.006	0.019	0.042	t=1.06	0.299
right UTM transversal	0.029	0.004	0.029	0.006	0.017	0.036	0.029	0.006	0.028	0.008	0.018	0.041	t=0.12	0.905
left UTM longitudinal	0.028	0.007	0.027	0.005	0.017	0.049	0.027	0.007	0.028	0.010	0.019	0.037	Z=0.83	0.422
left UTM transversal	0.027	0.004	0.027	0.006	0.020	0.036	0.027	0.005	0.026	0.008	0.018	0.038	t=0.38	0.709

Supplement Table 9. Comparison of migraine patients' UTM gray scale values of the innermost 75% of muscular tissue surrounding a reference point/mTrP. Reference point values were pooled for each side of the UTM. Gray scale values are given on a numeric scale from 0-255. * marks statistical significance with p<0.05, † marks statistical significance after correction for multiple testing. Abbreviations: SD standard deviation, IQR interquartile range, Min minimum, Max maximum, UTM upper trapezius muscle, mTrP myofascial trigger point, RP reference points

	**RP**						**mTrP**						**Test values**	
	Mean	SD	Median	IQR	Min	Max	Mean	SD	Median	IQR	Min	Max	*t/Z*	p
**Gray scale mean**														
right UTM longitudinal	42.30	11.23	42.50	8.90	16.71	60.99	36.82	11.73	35.46	20.54	18.03	53.92	*t*=1.35	0.209
right UTM transversal	46.26	11.36	44.10	14.54	25.41	65.55	33.89	11.79	33.07	10.53	14.84	59.32	*t*=3.32	0.009*
left UTM longitudinal	44.16	9.21	45.18	13.27	26.76	54.64	42.31	10.27	42.04	20.58	29.46	55.92	*t*=0.74	0.485
left UTM transversal	42.71	9.61	44.48	19.01	29.24	54.24	45.18	11.38	44.23	22.81	32.46	60.90	*t*=0.68	0.520
**Gray scale SD**														
right UTM longitudinal	22.82	2.87	22.88	3.36	17.92	28.38	20.22	4.70	19.76	7.00	13.73	29.76	*t*=2.10	0.065
right UTM transversal	25.79	3.57	25.22	3.88	20.03	32.10	20.49	4.59	19.54	5.16	14.69	31.22	*t*=5.20	<0.001*†
left UTM longitudinal	22.93	3.96	21.66	6.90	18.35	29.34	23.09	5.44	22.39	8.70	15.10	31.89	*t*=0.11	0.914
left UTM transversal	24.91	4.72	23.93	8.42	18.59	32.19	26.34	3.95	28.57	6.03	19.70	30.03	*t*=1.10	0.316
**Gray scale max**														
right UTM longitudinal	169.57	18.38	169.39	28.67	140.33	202.00	149.67	36.54	153.67	58.08	102.33	216.00	*t*=1.98	0.079
right UTM transversal	190.87	20.23	187.42	26.21	151.83	222.33	160.07	32.65	157.33	45.25	113.00	221.66	*t*=3.04	0.014*
left UTM longitudinal	160.97	22.55	150.71	34.17	131.00	198.33	167.79	37.05	178.50	66.92	107.33	212.33	*t*=0.67	0.525
left UTM transversal	180.89	27.95	184.50	49.83	134.50	213.92	187.76	25.21	196.67	46.00	146.33	212.33	*t*=0.75	0.482
